# An update of the long-term outcome of patients with nonspecific pleurisy at medical thoracoscopy

**DOI:** 10.1186/s12890-021-01596-2

**Published:** 2021-07-12

**Authors:** Yan-Xia Yu, Yuan Yang, Yan-Bing Wu, Xiao-Juan Wang, Li-Li Xu, Zhen Wang, Feng Wang, Zhao-Hui Tong, Huan-Zhong Shi

**Affiliations:** 1 Department of Respiratory and Critical Care Medicine, Beijing Institute of Respiratory Medicine and Beijing Chao-Yang Hospital, Capital Medical University, Beijing, 100020 China; 2 Department of Respiratory and Critical Care Medicine, Beijing Chao-Yang Hospital, Capital Medical University, 8 Gongti Nanlu, Chaoyang District, Beijing, 100020 China

**Keywords:** Nonspecific pleurisy, Medical thoracoscopy, Outcome

## Abstract

**Background:**

Medical thoracoscopy (MT) is recommended in patients with undiagnosed exudative pleural effusion and offers a degree of diagnostic sensitivity for pleural malignancy. However, not all patients who undergo MT receive an exact diagnosis. Our previous investigation from 2014 summarized the long-term outcomes of these patients with nonspecific pleurisy (NSP); now, we offer updated data with the goal of refining our conclusions.

**Methods:**

Between July 2005 and August 2018, MT with pleural biopsies were performed in a total of 1,254 patients with undiagnosed pleural effusions. One hundred fifty-four patients diagnosed with NSP with available follow-up data were included in the present study, and their medical records were reviewed.

**Results:**

A total of 154 patients were included in this study with a mean follow-up duration of 61.5 ± 43.7 months (range: 1–180 months). No specific diagnosis was established in 67 (43.5%) of the patients. Nineteen patients (12.3%) were subsequently diagnosed with pleural malignancies. Sixty-eight patients (44.2%) were diagnosed with benign diseases. Findings of pleural nodules or plaques during MT and the recurrence of pleural effusion were associated with malignant disease.

**Conclusions:**

Although most NSP patients received a diagnosis of a benign disease, malignant disease was still a possibility, especially in those patients with nodules or plaques as noted on the MT and a recurrence of pleural effusion. One year of clinical follow-up for NSP patients is likely sufficient. These updated results further confirm our previous study’s conclusions.

## Introduction

Pleural effusion (PE) is a manifestation of a variety of aetiologies, including diseases local to the pleura; underlying lung organ dysfunction, or systemic conditions; and drug use. It accounts for more than 125,000 hospital admissions per year in the United States, and inpatient cost estimates are greater than $5 billion per year [1]. The aetiological diagnosis of pleural effusion plays a decisive role in the treatment choice.

Medical thoracoscopy (MT) refers to the examination of the pleural space and has been well documented to be a highly sensitive and safe procedure for diagnosing exudative pleural effusions [2, 3]. Our previous study revealed that the overall diagnostic efficiency of MT was 92.6% (771/833) [4]. However, pleural fluid analysis and pleural biopsy failed to yield a definitive diagnosis after MT for a proportion of patients. The histological findings of the pleural biopsies in these patients are not specific, unlike with tuberculosis or malignancies, but instead show nonspecific pleural inflammation (pleuritis/fibrosis).

Our previous study summarized the long-term outcomes of nonspecific pleurisy (NSP) patients after MT before June 2014 in Chaoyang Hospital. The conclusion was that patients with NSP after MT should be closely monitored, especially those with a recurrence of pleural effusion during follow-up or with pleural nodules or plaques found during MT [5]. In this study, we updated these data to potentially refine our conclusions.

## Methods

### Patients

The present study protocol was approved by the institutional review board for human studies of Beijing Chao-Yang Hospital in Beijing, China.

MTs were performed in a total of 1,254 patients with undiagnosed pleural effusions between July 2005 and August 2018, and pleural biopsies were collected from the suspected areas and were systematically taken from several parts of the parietal pleura for mycobacterial, cytological, histological, and immunohistochemical examination in our institution. The detailed medical history, clinical presentation, laboratory examination results, and imaging data of the patients were also recorded [4]. However, only those data of patients with pleural effusion and nonspecific histological diagnosis of MT were included in the current study for a total of 172 eligible cases, and among these cases, complete follow-up data were obtained for 154 patients. Before MT, initial diagnostic workups, which included a detailed medical evaluation, pleural radiological assessments, pleural fluid analyses, and/or closed pleural biopsy examination, were performed in all patients, but their pleural effusions remained undiagnosed. The characteristics of the study population are presented in Table 1.

**Table 1:** Characteristics of study subjects with NSP (n = 154)

Variables	Values
Age, years, mean ± SD	61.8 ± 14.5
Sex, male/female, n (%)	98/56 (63.6/36.4)
Smoking status, n (%)	
Non-smoker	81 (52.6)
Current or previous smoker	73 (47.4)
Follow up duration (months)	61.5 ± 43.7
Diagnosis of pleural malignancy during follow-up	
Lung cancer	7 (36.8)
Mesothelioma	6 (31.6)
Gynaecological tumor	2 (10.5)
Breast tumor	1 (5.3)
Prostatic cancer	1 (5.3)
Plasmacytoma	1 (5.3)
Thymoma	1 (5.3)
Diagnosis of pleural benign disease during follow-up	
Tuberculosis-induced	24 (35.3)
Heart failure	16 (23.5)
Parapneumonic effusion	13 (19.1)
Connective tissue diseases	5 (7.4)
Pulmonary embolism	4 (5.9)
Pneumosilicosis	4 (5.9)
After splenic embolization surgery	1 (1.5)
After coronary artery bypass grafting	1 (1.5)

### Thoracoscopic procedures

MT was performed by thoracic physicians in our pulmonary procedural suite as described in a previous publication [4]. Pleural fluid and pleural biopsy samples obtained from each patient were analysed. Biochemical parameters, including concentrations of total protein (TP), lactate dehydrogenase (LDH), PE TP/serum TP, and PE LDH/serum LDH in fluid samples, were measured. Cytological examination of pleural fluids and histopathological and immunohistochemical assessments of pleural biopsies from all patients were performed.

### Diagnostic criteria for NSP

NSP was diagnosed if the histology report of the pleural tissue revealed reactive fibrous pleural thickening, fibrinous pleurisy, fibrosis, fibrous connective tissue, chronic inflammation, benign changes, or dense fibrous tissue in the absence of malignant pleural infiltration, granulomata, pleural vasculitis, or evidence of bacterial infection [6].

### Follow-up

The end of our follow-up period was August 1, 2020, and individual patients were monitored until this deadline or their death. During the follow-up period, the following information was gathered from the patients or their relatives every month by telephone or personal interview: (1) patient demographics; (2) the onset of any new symptoms, such as cough, chest pain, or shortness of breath; (3) recurrence of pleural effusion after MT; (4) any subsequent diagnosis of the cause of pleural effusion later in the course of treatment; and (5) when and how the subsequent diagnosis was established.

### Statistical analysis

All analyses were performed with the Statistical Package for the Social Sciences software version 25.0 (IBM Corporation, Armonk, NY, USA). Data are presented as the mean ± standard deviation values or numbers with percentages. Descriptive statistical methods were used for data analysis. The chi-squared test was used to compare categorical variables, while the Student’s *t*-test was performed to compare the mean values of two groups. All reported *p *values were two-sided, and effects were considered significant if *p* < 0.05.

## Results

Between July 2005 and August 2018, a total of 1,254 patients with undiagnosed pleural effusions successfully underwent MT, and pleural biopsy samples were obtained for diagnostic evaluation. Eventually, NSP was established as the final diagnosis in 172 patients, and 154 patients with available follow-up data were included in our current analysis. As shown in Table 1, 98 patients were men and 56 were women, with a mean age of 61.8 years, and the mean follow-up was 61.5 ± 43.7 months (range: 1–180 months).

**Fig. 1:**
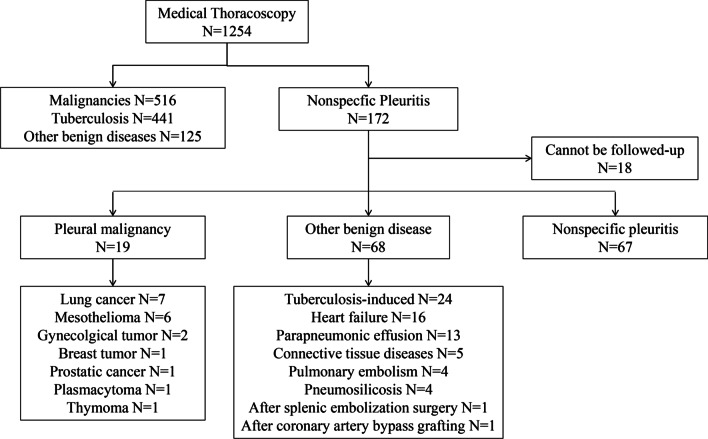
Eventual diagnosis of patients undergoing medical thoracoscopy

The final diagnoses are shown in Fig. 1. Nineteen of 154 patients with NSP (12.3%) were subsequently diagnosed with pleural malignancies (Table 2), including seven with lung cancer, six with mesothelioma, two with gynaecological tumors, one with a breast tumor, one with prostatic cancer, one with plasmacytoma, and one with thymoma. Additionally, 68 of the 154 patients with NSP (44.2%) were diagnosed with benign diseases, including 24 with tuberculosis, 16 with heart failure, 13 with parapneumonic effusion, five with connective tissue diseases, four with pulmonary embolism, four with pneumosilicosis, one after splenic embolization surgery, and one after coronary artery bypass grafting. In 67 of the 154 patients with NSP (43.5%), no exact cause of their condition could be determined. They were eventually diagnosed with idiopathic pleural effusion.

**Table 2:** Patients with Subsequent Pleural Malignancy

	Age	Sex	Smoking history	Previous history		Pleural fluid appearance	Pleural nodules	Pleural plaques	Subsequent pleural diagnosis	Subsequent diagnostic procedure	Time from NSP to cancer diagnosis (months)
				Asbestos exposure	Previous cancers						
Patient 1	F	70	Y	N	N	Serous	N/A	N/A	Mesothelioma	PNB	1
Patient 2	M	64	Y	N	N	Blood-stained	N	N	Mesothelioma	Second MT	4
Patient 3	F	52	N	N	N	N/A	Y	Y	Mesothelioma	Second MT	10
Patient 4	F	48	N	N	N	Serous	Y	N	Mesothelioma	OLB	8
Patient 5	M	77	Y	N	N	Serous	Y	Y	Mesothelioma	Liver-biopsy	5
Patient 6	F	76	Y	N	Kidney cancer	Serous	N	Y	Lung cancer	PFC	1
Patient 7	F	41	N	N	N	Serous	N	N	Thymoma	PNB	1
Patient 8	M	56	Y	N	N	Blood-stained	N	Y	Lung cancer	PNB	1
Patient 9	M	61	N	N	N	Serous	Y	N	Prostatic cancer	Prostate-biopsy	5
Patient 10	F	62	N	N	N	N/A	N/A	N/A	Lung cancer	PNB	5
Patient 11	M	76	N	N	N	Serous	N	Y	Plasmacytoma	BMA	1
Patient 12	F	62	N	N	N	Serous	Y	N	Lung cancer	PNB	1
Patient 13	F	43	N	N	Breast tumor	Serous	N	Y	Breast tumor	Chemotherapy for breast cancer	1
Patient 14	M	65	Y	N	N	Serous	N	Y	Lung cancer	PFC	2
Patient 15	F	71	N	N	N	Serous	Y	N	Gynaecological tumor	Endometrial-biopsy	2
Patient 16	F	58	N	N	N	Serous	N	Y	Mesothelioma	PNB	9
Patient 17	M	64	Y	N	N	Blood-stained	N	Y	Lung cancer	PFC	3
Patient 18	F	61	N	N	Breast tumor	Serous	Y	Y	Gynaecological tumor	Ovarian-biopsy	1
Patient 19	M	80	Y	N	N	Serous	N	Y	Lung cancer	Bronchoscopy	1

F, female; M, male; Y, yes; N, no; N/A, information not available; NSP, nonspecific pleurisy; PFC, pleural fluid cytology; PNB, percutaneous needle biopsy; OLB, open-lung biopsy; BMA, bone marrow aspiration

Sixteen patients with heart failure were enrolled in this analysis. According to Light’s criteria, 12 cases were exudative pleural effusions, and 4 cases were transudative pleural effusions. The 4 patients with transudative pleural effusion did not undergo thoracocentesis before medical thoracoscopic examination because the volume of pleural effusion was small. For the 12 cases with exudative pleural effusion, the pleural biopsy pathologies were neither tuberculosis nor malignancy. Combined with these patients' previous history of heart disease (hypertension in 12 patients, coronary heart disease in 5 patients, atrial fibrillation in 4 patients, rheumatic heart disease in 2 patients and hypertrophic cardiomyopathy in 1 patient) and the decrease in pleural effusion after standardized management of primary heart disease, we determined that these pleural effusions were the result of heart failure. The reason that these pleural effusions were exudates may be due to the use of diuretics.

As shown in Table 2, the group of 19 patients with false-negative diagnoses consisted of eight men and 11 women with a mean age of 62.5 ± 11.2 years. In this subgroup, 42.1% of patients had a smoking history, and only two patients had neither pleural nodules nor pleural plaques. The subsequent diagnostic procedures of these patients largely involved tissue biopsy; only one patient was diagnosed by the reduction of pleural effusion after chemotherapy for breast cancer. The average time from NSP biopsy to cancer diagnosis was 3.3 ± 3.0 months (range: 1–10 months).

Table 3 presents details of patient demographics, symptoms, abnormalities during microscopy, and biochemistry of the pleural effusion in patients with NSP. The results indicate that there was a higher likelihood of diagnosing pleural malignancies among patients with the recurrence of pleural effusion (*p* = 0.035), pleural nodules (*p* = 0.034), or pleural plaques (*p* = 0.038). No association was seen when considering other characteristics.

**Table 3:** Basic demographics of patients, symptoms, and pathology, microscopy and biochemistry results of pleural effusion of patients with nonspecific pleurisy

	Benign disease course (n = 154)	False-negative result (n = 19)	*p *value
Patient demographics			
Age, year (mean ± SD)	61.7 ± 15.0	62.3 ± 11.2	0.825
Male sex (%)	66.7	42.1	**0.037**
Smoking status (%)	48.1	42.1	0.621
Symptoms			
Cough (%)	45.1	47.4	0.853
Dyspnoea (%)	73.7	84.2	0.322
Chest pain (%)	22.6	10.5	0.229
Fever (%)	14.3	10.5	0.867
Loss of weight (%)	6.0	10.5	0.458
PE recurrence (%)	29.8	56.3	**0.035**
Macroscopic abnormalities found at thoracoscopy			
Nodules (%)	18.8	41.2	**0.034**
Plaques (%)	38.3	64.7	**0.038**
Thick adhesions (%)	50/154 = 32.5	6/19 = 31.6	0.938
Biochemical parameters in pleural fluid			
TP (mean ± SD)	44.5 ± 10.9	41.9 ± 6.3	0.337
LDH (mean ± SD)	287.5 ± 270.0	205.1 ± 125.6	0.232
PE TP/serum TP	0.67 ± 0.16	0.64 ± 0.11	0.598
PE LDH/serum LDH	1.44 ± 1.09	1.03 ± 0.62	0.203

The numbers in bold indicate the *p* values < 0.05, which mean the effects were considered significant

LDH, lactate dehydrogenase; TP, total protein

## Discussion

In respiratory medicine, pleural effusions are one of the most complicated conditions to diagnose [7]. MT is a minimally invasive method for diagnosing unidentified pleural effusions with a high rate of sensitivity of 85–100% [8–17]. However, not all patients undergoing MT may receive a definitive diagnosis. Nonspecific histopathology results can suggest the occurrence of a sampling error or genuine benign pleural disease [5]. In our study, a clear aetiological diagnosis was made in 56.5% (87/154) of undiagnosed cases after follow-up, including 19 patients who were eventually diagnosed with pleural malignancy.

Benign diseases continued to be the most common diagnosis during follow-up (68/87 patients; 78.2%). However, the atypical nature of pathology ensures that benign diseases are not easily detected by MT specificity. Even in the case of tuberculous pleurisy, the reaction occurring in the pleura may cause effusion in the absence of granulomatous inflammation [18]. Thus, the diagnosis of benign disease depends upon the patient’s clinical manifestation and the doctor’s empirical treatment.

Although thoracoscopy has a high diagnostic success rate for pleural effusion, it is still not a foolproof method. In our study, 19 of 154 NSP patients were subsequently diagnosed with pleural malignancy, including lung cancer and malignant pleural mesothelioma, which were most common. For patients 5, 9, 11, 13, 15 and 18, there was no direct evidence for malignancy in the pleural biopsy or pleural effusion analyses, but pleural effusion was found repeatedly in these patients during the follow-up. There was no other evidence for causes of pleural effusion except for the malignant tumors diagnosed in other regions. Therefore, we classified it as malignant pleural effusion. Most of the lung cancer patients were relatively easy to diagnose since chest computed tomography imaging is likely to accurately confirm tumors, and all of these individuals were diagnosed within five months. The more difficult disease to diagnose is pleural mesothelioma because its lesion depth makes it difficult to detect using MT. For patient 1, the short delay in the final diagnosis of mesothelioma may be due to a lack of adequate sampling during the MT, and she was finally diagnosed by percutaneous biopsy within a month. However, in comparing our previous [5] and current research, the rate of missed diagnoses for pleural mesothelioma decreased from 12.5% (5/40) before June 2014 to 5% (1/20) after June 2014. This progress can be attributed to the significant procedural advancements made since the first recorded thoracoscopies [19] and the ongoing improvements in immunohistochemistry and thoracoscopic techniques. For the histological diagnosis in the 6 mesothelioma patients, only 3 of them had mesothelial dysplasia. We attempted to find a potential 'precancerous' lesion in these patients, but the results were not satisfactory. Indeed, 5 of them had cellulose exudation, inflammatory cell infiltration, or fibrous tissue hyperplasia, but we did not think these lesions can be considered as potential 'precancerous' lesion. These lesions are symptoms of chronic inflammation, which can be seen in many diseases. We hope there will be more sophisticated research in this area in the future.

The factors that may increase the likelihood of underlying malignant disease in patients with NPS remain the same as those found in our previous study, including nodules or plaques in MT and the recurrence of pleural effusion. The presence of any of these lesions may have a marked impact on increasing the incidence of false negatives at thoracoscopy. Patients with these characteristics require close monitoring.

In terms of the follow-up time, our study had a mean follow-up time of 61.5 months, and the maximum time to the diagnosis of malignancy was 10 months. These results further confirm our previous conclusion that, for NSP patients, one year of clinical follow-up is likely sufficient to detect most cases of malignant disease. It should be noted, however, that during our follow-up period, we identified a pleural effusion patient who developed lung cancer two years after thoracoscopy. Since he was initially hospitalized with cardiac insufficiency, the pleural effusion was initially a transudate. The amount of pleural effusion was reduced following the treatment for cardiac insufficiency, so we considered the lung cancer diagnosed two years later to be a new emerging disease. As such, we did not consider his follow-up data in the design of our conclusions.

The causes of pleural effusion during follow-up could not be determined in 67 patients. Among them, we particularly focused on the 21 NSP patients who did not obtain a definitive diagnosis in our previous study. These patients had already undergone a sufficiently long follow-up period, and yet, their diagnosis did not change. This indicates that the conclusions made in our previous study are accurate and reliable.

In conclusion, MT is a useful tool for diagnosing PE. NSP can be diagnosed in a number of patients, and false-negative results can also occur. Although the diagnosis of most NSP patients is benign disease, malignant disease is also a possibility, especially in patients with nodules or plaques following MT and the recurrence of pleural effusion. This study further confirmed our previous study’s main conclusion that one year of clinical follow-up for NSP patients is likely sufficient. We look forward to conducting more multicentre prospective studies to explore the diagnosis and prognosis of NSP patients after MT.
